# No Common Ground: A Spatial-Relational Analysis of EU-China Relations

**DOI:** 10.1007/s11366-021-09769-w

**Published:** 2021-12-07

**Authors:** Katja Levy, Ágota Révész

**Affiliations:** 1 Manchester China Institute, University of Manchester, 178 Waterloo Place, Manchester, M13 9PL UK; 2 Center for Cultural Studies on Science and Technology in China, Technische Universität Berlin, Kaiserin-Augusta-Allee 104-106 (Raum 1110), 10553 Berlin, Germany

**Keywords:** EU-China relations, Foreign policy, EU China-policy, COVID-19 vaccine

## Abstract

It is no secret that EU member states cannot come to terms on a unified China-policy. Most studies on EU-China relations come to the conclusion that disagreement exists and that this fragmentation is utilized by Chinese foreign policy in a kind of divide and rule strategy. However, the question as to why the EU members disagree has not been answered satisfactorily. This paper investigates the reasons for this discord from the perspective of the core-periphery theory. We illustrate how the spatial position of nations within Europe–in a geographical and political sense–shapes their outlook on China. As a case study to illustrate the differences in the outlook on China of among EU member countries, we analyse the discourses on Chinese COVID-19 vaccines in the Hungarian and German press from April 2020 until summer 2021. We argue that these differences have their grounds in the spatial-relational positioning at either the core or the semi-periphery of the EU. Based on our findings we suggest that a sustainable EU China-policy has first to address these differences in foreign policy outlook and then find a common ground.

## Introduction

On 30 August 2017 then German Foreign Minister Sigmar Gabriel demanded that China stick to a “One Europe policy”. “If we do not succeed […] in developing a single strategy towards China,” he said, “then China will succeed in dividing Europe.”^1^ With these words he was alluding to China’s standard requirement for all its diplomatic partners to stick to the “One-China policy”, i.e. to acknowledge that the People’s Republic of China (PRC) represents China on an international level and that Taiwan and Tibet are legitimate parts of the Chinese nation and territory – and concluding that the EU can similarly expect the Chinese government to “pursue a one-Europe policy and not try to divide us”. He also alluded to the often repeated (Western) analysis of China’s foreign policy as a divide-and-conquer strategy, e.g. Stanzel as cited in Strittmatter [1].^2^ In other words, by insisting on bilateral relations with individual member states rather than on inter-regional relations with Europe as a unity of states, the PRC is presumed to have achieved better economic and other advantages with weaker individual countries than it could have done with a strong and unified European Union (EU) representing a market of 500 million consumers.^3^

For a long time, European politicians as well as observers of EU-China relations have been arguing in the same vein for a united China-policy by all EU member states. The argument has also been expressed at the highest EU foreign policy level: Josep Borrell, the EU’s High Representative for Foreign Affairs and Security Policy, said the following in March 2020 about China’s aid during the coronavirus crisis: “China is aggressively pushing the message that, unlike the US, it is a responsible and reliable partner. […] [W]e must be aware there is a geo-political component including a struggle for influence through spinning and the ‘politics of generosity’. Armed with facts, we need to defend Europe against its detractors”.^4^

Why the 27 EU member states cannot come to terms with a unified China-policy is the puzzle that this article wants to address. We argue that the way member states shape their China policies is deeply rooted in their individual histories and foreign policy experiences over roughly the past two hundred years, and their ensuing needs and resulting expectations towards the EU and China. We find that these differences often go back to the countries’ perceptions of their geopolitical positions, with a grouping appearing to form along differences and commonalities in those perceptions.

Understanding the differences among European member states in their relations to China (and other countries) is relevant. Knowing the reasons why member states’ governments have their individual specific perspectives on China can help the EU as institution make its China-policy more easily acceptable for its member states. Acceptance, again, is the basis for a more unified China-policy which enables the EU to act and negotiate independently and on equal footing with the other world powers China and the USA.

This article emphasises a historical-spatial explanation for the different perspectives of EU member countries on China. While the conclusions themselves regarding EU-China relations may not add much to existing literature on the topic, our contribution to the research lies in explaining this situation and in mapping the points that need to be tackled in order to achieve an EU China-policy that is truly supported by all its member states.

How will we meet this article’s objective of identifying one of the root causes for the seemingly unbridgeable differences among EU member states in their China-policy? Based on the core-periphery theory explicated in the section on theory below, we start from the following proposition:

EU member states cannot reach a unified China-policy because of fundamental differences in outlook regarding the EU as a community and China as a counterpart in foreign policy.

To provide evidence for this proposition we conduct a two-step qualitative analysis in two case countries. We selected Germany and Hungary as the cases because they occupy relatively extreme positions on the core-periphery map of Europe. We do not claim that they are representative for other countries in all aspects.^5^ Instead, we wish to highlight their differences along the core-periphery binary. Germany is an example of a core country and Hungary of a semi-periphery country in Europe. For both we first analyse why they view themselves as a core or periphery country from a historical perspective. We then pursue the two cases by conducting a qualitative textual analysis of recent media and political discourse on Chinese vaccines against Covid-19.^6^ We thereby illustrate their respective different outlooks on China as a result of their spatial positioning and self-reflection within Europe and vis-a-vis China. By demonstrating that the somewhat extreme examples of Germany and Hungary show such fundamental differences in their perspectives, we want to provide a new approach to an EU China-policy that accommodates the different starting points and interests of its members.^7^ This initial study cannot explicate the positions of other countries in Europe but we hope to show that analysing the positions of each country within Europe can help to explain their outlooks on China and how this knowledge can be used as a basis for sustainable foreign policy in this area. As two China researchers who wish to help build a sustainable, respectful and peaceful relationship between Europe and China, our focus is on the EU member states and their relations to China. However, this approach might also prove helpful in studying EU foreign policies regarding other countries and regions of the world.

We proceed as follows: in the next section we explain why and how the core-periphery theory forms the theoretical framework for our study. The third section is a two-part, two-country case study. The first step of the analysis is historical with a brief outline of the factors that led each country to perceive its particular spatial positioning in Europe. These perceptions are of course linked to the respective geographic positions, but go beyond them in light of shifting borders, neighbourhood constellations and coalitions. In the second step we systematically analyse German and Hungarian discourses on COVID-19 vaccines originating in China in the national press of each country and public speech by their political elites. We thereby illustrate great differences in the countries’ outlooks towards China. The fourth and final section summarises our findings and draws some conclusions about possible reasons for divergent China-policies within the EU.

## Theoretical Framework—Core and Periphery in Europe

In examining variations in EU member states’ outlooks on China and possible reasons for these variations, we are informed by two strands of research: studies on variations in economic and political integration among EU member states, and studies on variations in their China-policies.

There have been many attempts to explain differences among the member countries of the EU. Jürgen Habermas and Jacques Derrida are prominent proponents of a *Europe of different speeds*. They observed that only the “core European nations” were ready for a common foreign policy, common security policy, and common defence policy. In their opinion, the “core of Europe” [they speak of “old Europe”] had to march first, because “[o]nly such a step will succeed in generating momentum that other member states—initially in the Euro zone—will not be able to resist in the long run.” They called for avoiding separatism between core and periphery, but only a difference in speed [6]. Timothy Garton Ash took the European financial crisis to illustrate imbalances among the EU member states and explained these by means of institutional flaws within the EU [7]. Vivien Schmidt proposed a way to deal with these differences in interests and political attitudes by employing what she calls a *soft-core Europe* in which the member states would exercise their decision-making in issue-related clusters instead of having to vote unanimously on every single issue [8].

Researchers have also sought to explain the differences in China-policy among EU member countries and even among the different bodies of the EU [9]. A very concise attempt was the “China Power Audit” published by the ECFR think tank in 2009. This report^8^ categorised EU member states along two main dimensions: how they managed China’s impact on the European economy and how they engaged China politically [10]. It identified four clusters of countries with similar attitudes toward China: the “assertive industrialists”,^9^ the “ideological free-traders”,^10^ the “European followers”,^11^ and the “accommodating mercantilists”.^12^ Their China-policy approaches range from great engagement for economic benefit to strong commitment to human rights, plus varying combinations thereof. Although this report seems outdated today because Europe and China have changed dramatically, it is worth mentioning as one of the first attempts to categorise the China policies of different member states. In a second report the ECFR refrained from updating the four clusters [11]. In the eight years between the first and the second ECFR reports, China established the Belt and Road Initiative and the China-CEE Initiative.^13^ It also undertook a shopping spree of high-tech and other companies in Europe that culminated in the acquisition of the Kuka robotics producer by the Midea company in 2016. This purchase provoked Germany and Europe to establish investment screening procedures to prevent further buy-outs of European high-tech companies and core infrastructures. These events alone illustrate how fast European-Chinese relations have developed and changed in quality. Differences in the reports also show that to understand countries’ attitudes towards China, an approach is needed that takes the economic, political and historical origins of the different perspectives into account.

A recent report by the European think-tank network on China (ETNC) suggests that EU member states may be grouped into four different types with respect to the soft power strategy applied by China, ranging from a “no soft power” strategy due to lack of interest in China in countries such as Hungary, to damage containment efforts in e.g. Italy,^14^ to struggling with growing vigilance in e.g. Germany, to a “state of free fall” in countries such as Czechia and Sweden [16]. An earlier report by the same network observes that the attitudes of EU member states toward China are shaped by the three factors of historical legacy, economic relations, and Chinese pressure [17]. Regarding the impact of historical legacy on bilateral relations between EU member states and China, the report states that “older democratic institutions tend to resist […] downgrading [of the importance of the values democracy, human rights, and rule of law] more than those in younger democracies” [17]. The authors also find that “[p]olitical actors that are critical of the EU tend to use China as leverage vis-à-vis the EU institutions and other EU member states [17].

To get to the roots of variations in China-policy among EU member states, we suggest looking at the differences among these states in general and their attitudes towards China in particular as two sides of the same coin, and combining the respective approaches.

A group of scholars whom Weissenbacher assigns to the “European dependency school”^15^ reformulate and redefine the basic propositions of dependency theory^16^ or Latin American structuralism to explain variations in development among EU member states. Their basic idea is that social and political-economic conflicts can be expressed along territorial/spatial lines [18]. This approach indicates that expectations of divergent development (“trickle-down effect”) among EU member states proposed by economic models informed primarily by neoliberalism would not materialise due to fundamental flaws in the institutional conception of the EU [7, 18].

Using the core-periphery approach as a conceptual framework and a model of income distribution in combination with a measure of industrialisation, Weissenbacher introduces a core /semi-periphery / periphery typology for analysing EU member countries’ development status. He shows that from the 1960s until now, EU member states can be placed in three different groups according to their income and industrialisation status, and that this typology is also in line with their spatial distribution in Europe from the core to the semi-periphery to the periphery, albeit with some countries hovering between adjacent types, e.g. from periphery to semi-periphery and back or from semi-periphery to core and back [19]. According to this typology, Germany^17^ has always belonged to the core of the EU and Hungary always to the periphery since becoming a member in 2004 [19]. We prefer, however, to define Hungary’s position as semi-periphery, and use Kozma’s definition of “semi-periphery” here: “Semi-periphery is a group of ‘economies on the road’. On the one hand, those who are in a relatively favourable position in the periphery, striving to reach the position of the centre, and on the other hand, those who drift downwards from the bottom of the centre towards the periphery. […] If the struggle ceases, the economy concerned is irreversibly discharged into the periphery in a very short time. Therefore, the most characteristic feature of the semi-periphery is not the intermediate position, but the struggle against sinking into peripheral existence” [20].^18^

We argue that location in the core or periphery may have a profound impact on (and therefore explicatory value for) the outlook towards China because it determines how EU membership is perceived overall by the respective country. In short, we propose that the farther a country is from the core, the less its China-policy is aligned with that of the core because its likelihood of being dissatisfied with this position within the EU is greater. Or, to put it the other way around, the nearer a country perceives itself to the core of the EU, the more likely it is to have a critical policy outlook towards China. We distinguish here between China-policy including criticism of human rights, trade and other policies on the one hand, and trade and investment relations on the other which seem to run along other lines.

In the next section we use two case studies to illustrate how core or periphery positioning influences China policies in two EU member states.

## Case Studies

As a two-part illustrative case study we looked at the discourse on Chinese COVID-19 vaccines in 2020 and 2021 in Germany and Hungary. The two countries were selected because they exemplify two ends of Weissenbacher’s core / semi-periphery / periphery typology [19], with Germany belonging to the core group and Hungary the semi-periphery group of the EU. We are aware of the fact that there are significant differences among the countries within the core as well as within the (semi-)periphery, and we by no means wish to play down these differences. Yet we see a link between the relative position of these two countries and the way they frame and formulate their policies regarding the Chinese vaccine – and we believe it can point the way to future, more substantial research.

The recent discourse about Chinese vaccines against COVID-19 was selected because it exemplifies the significantly different attitudes of EU countries towards China. Before presenting the two cases, we sketch the background to the COVID-19 vaccines.^19^ China, Germany and the US were among the first countries to announce they had experts working on different kinds of vaccines. In the end it was Russia that presented its vaccine with the evocative name of Sputnik V in August 2020. Shortly thereafter, other countries presented their vaccines, including China in late 2020. In May 2020 President Xi Jinping announced at a World Health Assembly meeting that China considered its COVID-19 vaccines to be a “global public good”. The Chinese COVID_19 vaccines, primarily Sinopharm and to a lesser extent CoronaVac (produced by Sinovac) and Convidecia (manufactured by CanSinoBiologics) [26], have been shipped to eighty countries. Fifty-three of these countries (mostly developing nations) received it for free, while twenty-seven middle-income countries had to pay for it [27]. Unsatisfied^20^ with the slow pace of vaccination roll-out, shortages in supply, and delivery bottlenecks within the EU, several EU members turned to China (and Russia) for more vaccine supplies. Russia’s Sputnik V slowly won the acceptance of politicians across Europe, including in Germany. However, Chinese vaccines have not been approved by the European Medicines Agency (EMA) and their acceptance is limited. Central and Eastern European non-EU member countries, such as Serbia, Turkey, Belarus, Bosnia and Herzegovina, Montenegro, North Macedonia, and Ukraine have bought or are planning to buy Chinese vaccines. CEE state Hungary has bought 5 million doses and both Czechia and Poland are considering doing so [26, 29].

In the following we show how this foreign policy strategy was discussed in the two case-study countries of Germany and Hungary. By adding a historical background, we illustrate how the two countries came to be at the core (Germany) and the periphery (Hungary) of Europe and how this affected their respective views and policies on Chinese COVID-19 vaccines.

### Case 1: Germany

This analysis begins with the historical section and is then followed by the textual analysis.

#### Germany’s Position at the Core of Europe—From a Historical Perspective

West Germany and the subsequent reunited Germany are an exemplary case of a core country in Europe in line with the core-periphery theory. When the European Union was founded (initially as its predecessor organisation the European Coal and Steel Community) Germany had very strong incentives to become part of a peaceful post-war community of countries in Europe. It needed to re-establish trustful relations with its neighbours. Reflecting on the Nazi period and learning from historical mistakes remain a core element of “Germany’s self-identification and ha[ve] a big impact on its policy regarding democracy, the rule of law and human rights” [17]. In addition, West Germany needed the support of France, the United States and the Soviet Union to pursue its long-term goal of reunification. This led Germany to become a major protagonist and proponent of the EU. With its main goals of reintegration and reunification achieved, its motivation for supporting the EU then changed. As an export country, it benefitted in large part from the EU. Due to the unified stable currency regime, EU member states cannot devaluate their own currencies to be more compatible. At the same time, Germany’s goods are in high demand within Europe [7]. Germany found itself primarily in precisely the opposite situation from countries at the periphery of Europe, particularly in the south and the east, which were struggling especially following the financial and economic crisis of 2008–2009 [18]. Germany is not only situated in the geographical middle of the EU but has been a strong integrating and shaping force in Europe.

#### Germany and China

Over the years Germany has also developed strong and rather close relations with China. When the People’s Republic of China (PRC) was founded in 1949, post war-Germany was in the process of becoming divided. The German Democratic Republic (GDR) was founded a few days after the PRC, and followed the Soviet Union closely in its China-policy until the early 1980s. Diplomatic relations between the GDR and the PRC were established almost immediately, in 1949. Years of close exchange and economic cooperation followed until the Chinese-Soviet split and the Cultural Revolution, which also meant the end of relations between the PRC and the GDR. At the beginning of the 1980s the GDR slowly signalled its renewed interest in intensifying bilateral relations with China, although the Soviet Union waited much longer to begin normalising its relations [30]. The Federal Republic of Germany, on the other hand, was bound in its foreign policies to the Western world. Diplomatic relations with China were established in 1972, following Washington’s turn to China in the early 1970s in an effort to outflank the Soviet Union in the Cold War. Economic interests played an important role from the start. However, before Germany was unified it always had to bear the divided city of Berlin and the possibility of reunification in mind and shaped its foreign relations accordingly. The breakdown of West German-Chinese relations after the 1989 crackdown on the democracy movement coincided with the fall of the Berlin Wall, and reunified Germany waited until 1992 to resume the relationship. Germany’s China-policy since the end of the Cold War has always been shaped by three partially contradictory factors: economic interests, human rights concerns and the specific China-policy of the incumbent German leadership. Helmut Kohl, the chancellor during the unification process, was open to trade and investment relations. With the support of the minister-president of the state of Bavaria Franz Josef Strauß, he laid the foundation for the recent close German-Chinese relations. Gerhard Schröder, Kohl’s successor and chancellor from 1998 to 2005, explicitly courted China and Russia, which enabled economic relations to flourish. At the same time a particular form of dialogue policy was established leading to approximately eighty different bilateral dialogues today including annual government consultation rounds. Angela Merkel has had a different policy style, less pal-oriented but equally keen on maintaining good economic relations, coupled with a certain reserve towards the socialist government. Today, the two countries have a solid and complex economic relationship. Germany has transferred a large part of its production to the PRC and benefitted for many years from the huge market.

#### The Debate on Chinese Vaccines in Germany

For this frame analysis a German textual corpus was compiled consisting of media articles and parliamentary documents. The media articles are from the Genios database^21^ and comprised mainly (online) press articles and a few items from radio and TV. The analysis starts with the first mention of COVID-19 vaccinations in April 2020 and ends in June 2021. To be included in the textual corpus, the articles had to deal substantively with COVID-19 vaccination and include the keyword ’China’. In addition, documents from the German Bundestag, i.e. speeches, parliamentary motions, inquiries by MPs and answers from the government on issues related to the Chinese vaccine were included. After clean-up, the textual corpus for the German case study consisted of fifty-five articles from German press and news portals and thirty-six parliamentary documents.^22^

All texts were analysed for whether they contained specific frames for Chinese vaccine-related contents. Following the definition by Price we looked for “textual attributions of a message (including organisation, selection of content, or thematic structure) [that] render particular thoughts applicable, resulting in their activation and use of evaluations” [31].^23^ Regarding the framing of press articles on Chinese COVID-19 vaccinations, we made two main observations: (a) only general news articles framed the issue while specialist papers^24^ focused on reporting facts and strictly refrained from any framing or political assessment thereof; (b) from these non-specialist papers in our German media corpus we identified five major frames (Table 1): (1) competition in vaccine development and application in the race to become the world’s leading power; (2) a ruthless Chinese government that follows its own power goals without regard for victims at home or abroad; (3) China’s vaccination imperialism, i.e. use of its vaccine to suppress other peoples and/or extend its power in other countries; (4) China as an actor that cannot be trusted; and (5) China being ineffective or clumsy in shaping its international image (“Public Relations Failure”). Within these five general frames we distinguished nineteen sub-frames. The second part of the German textual corpus, namely the parliamentary texts, contains slightly different sub-frames and weighting. We found matches in the two corpora for Frame 3 (China Exercises Vaccination Imperialism, Frame 2 (Ruthless China), Frame 1 (The Vaccination Competition is a Competition for World Leadership), and Frame 4 (China Cannot Be Trusted).^25^ Two additional sub-frames were identified. However, Frame 5 (China’s Public Relations Failure) was not echoed in the parliamentary texts.

**Table 1:** The five frames in Germany’s media and parliamentary discourse on COVID-19 vaccines from China

Frame 1: The vaccination competition is a competition for world leadership	Frame 2: Ruthless China
**• The nation that is most successful in development, application and distribution of the vaccine has the potential for world leadership** • China frames itself as a responsible power by supplying vaccines to countries that cannot afford the expensive and scarce vaccines produced in Western (liberal) countries	• China cares more about its international image than its own population • China provides vaccines of lesser quality to its partner countries • China provides low quality medicine/vaccination to its own people • China has contributed considerably to the worldwide spread of COVID-19 because it has valued a positive image by suppressing free speech at home over informing the world of the virus • China uses a price-dumping strategy to outpace competing Western (liberal) vaccine suppliers • China tests its (inferior) vaccine on volunteers in other countries • It is doubted whether China plans to vaccinate its own population at all • China tried to benefit from the crisis (and the misery of others) **• China withholds vaccine from Taiwan due to political reasons**

Frame 3: China exercises vaccination imperialism	Frame 4: China cannot be trusted	Frame 5: China’s public relations failure
**• China offers its vaccine to third world and emerging countries as well as Central and Eastern European countries in order to increase its power, not for humanitarian reasons** • Some countries approve Chinese vaccine despite its unproven quality • China exploits the limited vaccine access in poor or outlawed^a ^countries to increase their dependency on China • China offers vaccines in order to exert political pressure onto recipient countries	• China is non-transparent • Information from China is either propaganda or lies • Chinese pharmaceutical companies are corrupt • Given vaccine scarcity, even German politicians suggest considering approval of Chinese vaccines despite doubts about their quality **• Chinese product quality (including vaccine quality) cannot be trusted**	• China uses vaccinations to improve its image in the world, but is not convincing • China makes the ridiculous claim that Western vaccines may have dangerous side effects

Normal text = frames from media articles; bold text = frames from parliamentary documents; underlined normal text = frames that appeared in both media and parliamentary texts

^a^“Outlawed” here means excluded by Western liberal countries from cooperation in the fight against the virus because of authoritarian or populist governments

These frames varied in frequency. Some were applied simultaneously. They also alternated over the period under investigation. We could not identify a clear shift in framing during the period of investigation, but the following development was evident: in the beginning discussion focussed solely on who would develop a vaccine first and what this would mean for world power relations, and subsequently on who would achieve herd immunity first by vaccinating their population and who would take the lead in helping poor countries which had thus far lacked access to the vaccines.

Discourse on different Chinese COVID-19 vaccines in Germany went through three phases. The first was the “vaccine development phase”, which lasted largely from spring to summer 2020. It focussed on which country would develop the first effective vaccine. The second phase, i.e. the “vaccine roll-out”, and the third phase, i.e. “vaccine sharing”, started simultaneously (in summer 2020) and are still underway at the time of writing (summer 2021) but have differed in intensity. The frames for this topic did not follow this time line but rather showed waxing and waning intensities.

Chinese COVID-19 vaccine development, roll-out and distribution is discussed by the German public as an issue of economic stability, secure ties to the Chinese market and rather secure prospects of receiving enough vaccine from the EU.

#### Frame 1: The Vaccination Competition is a Competition for World Leadership

China was the first country to allow emergency use of a domestic vaccine, namely in June 2020.^26^ However, it was Russia that stepped forward with the first fully developed and officially approved vaccine Sputnik V in August 2020. The German-US Pfizer-BioNTech vaccine applied for approval in November 2020. The US-American vaccine Moderna and the British vaccine AstraZeneca followed shortly thereafter. German media discourse in this initial phase of vaccine development was not yet extensive. News reports were dominated by Frame 1: “The Vaccination Competition is a Competition for World Leadership”.

It was accompanied by the idea that the winner of the race to the first vaccine would also gain a leading position in the world.

While research is being conducted around the world, U.S. President Trump is turning the medical challenge into a race with rival China […] The vaccine race between Trump and Chinese President Xi Jinping is headed for a stalemate, immunologist and author Laurie Garrett predicts in the New Republic: “In the end, it could be that these two power-hungry egomaniacs share the Nobel Peace Prize.”^27^

If China wins the global race for a vaccine, it will carry symbolic weight, experts judge. Especially for the U.S., China’s biggest geopolitical rival, this would be a defeat.^28^

Other articles emphasised that China was trying to present itself as a responsible actor in world politics, leaving it open as to whether the strategy was convincing.

In one article, an expert is quoted on China’s vaccination roll-out in Indonesia:

Emerging economies like Indonesia are undecided about which camp to choose in the conflict between China and Western countries. It is with them that the People’s Republic is trying to score an important point by presenting itself as a responsible partner.^29^

However, after China was the first country to come up with a vaccine, albeit only on an emergency approval basis, and Russia, another non-Western country, became the first to have a fully approved vaccine, framing shifted from the significance of speed to the significance of quality.

In parliamentary debates, the “competition for world leadership” frame was referenced in debates on the geopolitical dimension of vaccine distribution. Andrej Hunko (MP, The Left), for example, pointed out that China and Russia were much quicker than the EU in helping non-EU members in Europe, such as the Republic of San Marino and Serbia, in their quest for a vaccine.^30^

#### Frame 2: “Ruthless China”

In the next phase of vaccine roll-out, Frame 2 or “Ruthless China” came into play. Some articles accused China of caring more about its international image than its own population:

At present, it seems more important for Beijing to position itself as a helper in times of need and a global vaccine supplier. No criticism of this is voiced among its population.^31^

Referring to earlier product scandals in China, the issue of low-quality pharmaceuticals was often raised in the context of Chinese domestic vaccination campaigns as well as in articles about China providing vaccines to other countries.

These days, we can observe the kind of unconventional measures that the historic race for a Corona vaccine brings forward. The state oil company Petro China has made an immoral offer to its employees who plan to go on a business trip abroad: they may already be administered a promising vaccine as volunteers—without any clinical testing procedures. However, these guinea pigs were apparently not informed about possible side effects.^32^

But in mid-January, doubts arose about the Sinovac substance; according to tests, it had only about 50 percent efficacy in Brazil. In Indonesia, it was said to have been 65.3 percent in a smaller study. And so it hardly inspires confidence that companies like Sinovac and Sinopharm are keeping their test data largely under wraps for scientists in other countries. Nevertheless, CoronaVac has now been approved in both countries.^33^

China’s efforts to test vaccines in other countries were also subject to this line of reasoning.

In terms of vaccine development, China had been dealing with a luxury problem since the summer: The risk of infection had dropped to a low level after the lockdowns and the closing of the borders. For research, this was a disadvantage. It was not possible to find out whether a vaccine actually protects against infection. Chinese scientists therefore began testing their active ingredients in more than a dozen other countries, such as Peru, Argentina, Brazil, Bahrain, Morocco, Saudi Arabia, Indonesia, Turkey and the United Arab Emirates.^34^

Other sub-frames which cannot be cited due to space limitations include: China has considerably contributed to the worldwide spread of COVID-19 because it valued maintaining its good image by suppressing free speech at home over informing the world of the virus.^35^ China is also reported to engage in price dumping to outpace competing Western (liberal) vaccine suppliers.^36^

Interestingly, some journalists even doubt whether China has serious plans to vaccinate its own population at all:

While many countries want to vaccinate as large a proportion of their population as possible to achieve herd immunity, information about China’s vaccination policy for its own country is sparse. It is true that medical personnel and the military are reportedly to be vaccinated. But whether China also wants to achieve herd immunity for its approximately 1.35 billion citizens is not clearly communicated. The country has hardly recorded any new infections. Most of the Sinovac doses could therefore be destined for export.^37^

In parliamentary debates, the “Ruthless China” frame was used mainly in the context of vaccine deliveries for Taiwan which were reportedly obstructed by mainland China.^38^ The Liberal Democrat MP Alexander Graf Lambsdorff, for example, asked the state secretary of the Federal Ministry of Foreign Affairs in a parliamentary Q&A session:

Are media reports true that the government of Taiwan has asked the federal government for help in procuring and/or supplying SARS-CoV-2 coronavirus vaccine, and does the federal government, like the governments of Japan and the United States, plan to provide SARS-CoV-coronavirus vaccine to Taiwan in the short term?^39^

#### Frame 3: “China Exercises Vaccination Imperialism”

The third phase of sharing vaccines with the developing world runs in parallel to the second phase. However, the German media’s main concern here is China’s intentions regarding other countries. In the context of the COVID-19 vaccines global access initiative (COVAX) to facilitate equitable vaccine access for poor countries, for example, Frame 3 is that of China’s “vaccine imperialism”.

China is suspected of distributing its vaccines to developing countries under the pretext of humanitarian aid, but in reality with the primary aim of expanding its global power.

This view is advanced in the media:

Secretly China baits the world with its vaccine. […] China does nothing purely for humanitarian aid.^40^

While EU countries struggle for sufficient vaccine, Russia and China seem to distribute their products generously around the world. In this way, they secure influence—often to the detriment of Europeans.^41^

A similar view is voiced in a parliamentary motion by the Alliance 90/The Greens parliamentary group:

The gap left by the EU in the distribution of vaccines is currently being filled in part by China and Russia. These actors are not only concerned with the urgently needed supply of vaccines to the population, but also with creating financial and political dependencies. The EU and the German government are not doing enough to counter this, giving the impression that they are not taking the fears of their partners worldwide seriously enough.^42^

In the same vein we found articles on recipient countries approving insufficiently tested Chinese vaccines or production licenses thereof.^43^

The two remaining frames, namely Frame 4 “China Cannot Be Trusted” and Frame 5 “China’s Public Relations Failure”, are present in all phases of reporting on China’s vaccine development, roll-out and distribution.

#### Frame 4: “China Cannot Be Trusted”

Frame 4 also plays a role in some parliamentary documents. For example, the German government answered diplomatically to an inquiry by The Left party about approval of Chinese vaccines in Germany as follows:

The German government has repeatedly stated that it does not support the marketing of a vaccine without regulatory approval in Germany. Proper approval is important for confidence because, on the one hand, vaccines are used in healthy individuals and, on the other hand, quality, efficacy and safety must have been carefully tested and confirmed by an official procedure. These are also decisive criteria for the population’s quick and comprehensive acceptance of the offer of a vaccination. Therefore, the German government welcomes the start of application processing for the Russian vaccine Sputnik V at the European Medicines Agency with the aim of obtaining a European marketing authorization. The path of applying for European approval is also open to the Chinese vaccine manufacturers.^44^

#### Frame 5: “China’s Public Relations Failure”

However, Frame 5 about “China’s Public Relations Failure” was confined to media articles, such as ridiculing China’s purchasing strategy and domestic reporting:

By the way, China has also ordered from the class enemy: The People’s Republic has ordered 100 million doses from Biontech/Pfizer, but pro-government media are also reporting extensively on alleged side effects of the vaccine.^45^

#### Summary of the German Case Study

This brief sketch of media reports and parliamentary debate on China and its COVID-19 vaccination policy against the background of Germany’s historical positioning at the core of Europe, shows that the German media and political elite are generally highly suspicious about the intentions of the Chinese government. Even apparently humanitarian activities are conceived as acts of imperialism or crime. Even Chinese intentions regarding the health and safety of its own people are cast in doubt. The major frames in Germany’s media discourse are largely echoed in the parliamentary debates and motions. The only view not taken up by the politicians is that the Chinese government has failed in its public relations strategy. The above analysis shows a distanced, critical perspective on China and its vaccine policy. The journalists and politicians cited evidently do not see their country in a situation potentially requiring goods from or cooperation with China in the context of COVID-19 vaccination at some point. This is the perspective of Germany, a powerful country located at the core of Europe. What about Hungary, a country at Europe’s semi-periphery?

### Case 2: Hungary

For the Hungarian textual corpus, we have selected speeches by and interviews with Prime Minister Viktor Orbán (23 items), parliamentary addresses (43 items) and coverage of the Chinese vaccine in mainstream news media (mostly articles and occasionally videos, altogether 72 items). The latter group includes the six largest Hungarian news portals (24.hu, index.hu, origo.hu, HVG.hu, telex.hu, 444.hu)^46^ and the four leading political dailies which also have online editions (*Népszava*, *Magyar Nemzet*, *Magyar Hírlap*, *Világgazdaság*). Orbán’s speeches and interviews are usually also media appearances, such as his weekly interviews with *Kossuth Rádió*, the most popular news radio broadcaster in the country.^47^ Orbán dominates Hungarian media discourse not only because his speeches are widely broadcast across the Fidesz-controlled^48^ outlets with minor alterations—a survey prepared by Mérték Media Monitor Nonprofit Ltd. found approximately 80% pro-government media in Hungary^49^—but also because he exercises a strong agenda-setting influence even in oppositional media. The time span was from August 2020 to mid-March 2021. As the intention was to embed mention of the Chinese vaccine into the broader context of EU-Hungary relations, we also searched for the terms “West”, “East”, “Brussels” and “periphery” in addition to “Chinese vaccine”, “European Union” and “China”.

For Hungary, of the five main frames we identified, three (1–3) were used almost exclusively by the government (Orbán himself, Fidesz MPs or pro-government media), while the other two (4–5) were characteristic of discourse by the opposition. Given that approval of the Chinese vaccine was a key political issue in Hungary, these frames were employed intensively in parliamentary as well as media discourse (this also applies to frames by the opposition, the only two sub-frames missing from parliamentary addresses being the “China is suspect“ and “the Chinese vaccine is dangerous” ones) (Table 2).

**Table 2:** Frames in Hungary on the Chinese COVID-19 vaccine

Frame 1: Disadvantaged Hungary	Frame 2: Declining Western (Core) Europe—rising CEE
• Brussels is too slow, and Hungarians are dying because of this slowness • Brussels is not trustworthy—you can never tell what will come from there • Hungary should not demonstrate allegiance via the vaccine • For Brussels money is more important than time • Hungary can only rely on itself	• Countries outside the EU are doing well • You need a strong stance (not just rely on the West) • Pragmatism is superior—it also means relying more on the East • Brussels is blind and inward-looking, while the others can assess priorities better • Hungary has the best EU vaccination rate

Frame 3: China as opportunity	Frame 4: China cannot be trusted	Frame 5: Corrupt business between China and the Fidesz government
• The Chinese are fast and punctual with their vaccine shipments • The Chinese have known the virus longer than anyone else (they have experience) • Hungary will get its old life back sooner • Trust in the “traditional” Chinese vaccine method • EU-wide competition for the Chinese vaccine • Hungary’s Chinese vaccine procurement has saved many lives	• China is non-transparent (more specifically: its testing of the vaccine is non-transparent) • Vaccine documentation sent by China is incomplete • China is suspect • The Chinese vaccine is obsolete • The Chinese vaccine is dangerous • Even Fidesz supporters reject the Chinese vaccine • Only vaccines approved by the EMA can be trusted	• An authoritarian rule was needed for vaccine authorisation • “Vaccine conspiracy”: Hungarian and Chinese companies pursue joint corrupt deals for private profit • People’s safety is of no concern to the Fidesz government • Both the Hungarian and Chinese authorities are lying / governmental disinformation on the vaccine • The Fidesz government is playing the role of saviour • The Fidesz government is blackmailing the society with the Chinese vaccine (if it doesn’t get approved, we won’t get our old lives back for a long time)

Normal text = frames from media articles; underlined normal text = frames that appeared in both media and parliamentary texts

#### Historical Background

[T]he countries of West Europe have been the ’core’ of the world system as the most advanced and powerful countries of the world. They almost always dominated their neighbours to the East which by the twentieth century were generally very small and, at most, semi-developed countries. Furthermore, these small countries (although many of them were packed into the Habsburg empire for several centuries) suffered from the pressures of Western modernised and industrialised states on one side and the Eastern empires (Russian and Ottoman) on the other. They have been swinging through history between long waves of Westernisation and Easternisation. After the last five decades of Easternisation, there appears once more to be a fundamental turn in the other direction and so their Westernisation begins again [32].

Hungary is a pertinent case of the power struggle between East and West—and this binary still has a formative power in today’s politics. This non-Slavic nation views itself as having Asian nomadic ancestors who found their way to the Carpathian basin. After initial raids further into the West, in 1000 CE they opted for settled, agricultural statehood and conversion to Christianity to seal their integration into Europe. As the narrative goes, Hungarians have found their home in the “heart of Europe”—this “heart” was, however, close enough to the East to be devastated by the Mongols (1241-42), fall prey to the Ottoman Empire and become separated from the rest of Europe. Ottoman rule (1541-1699) was finally broken with Habsburg assistance, and the country was integrated into the Habsburg Monarchy, which was then reorganised under the name of the Austro-Hungarian Monarchy after the failed Hungarian independence movement of 1848-49. The Habsburg period (from the late seventeenth century to the end of World War I) positioned Hungary on the semi-periphery.

We can talk about Hungary’s semi-peripheral position not just in economic [20, 33–36] but also in cultural-discursive terms [37–43]. In parallel to bitterness from a sense of being “colonised” and wedged between East and West (as shown by the notion of Hungary being a “ferry nation”)^50^, there has also been a struggle for cultural “upward mobility” towards the core, as demonstrated by the discourse of an entire generation of intellectuals calling themselves “Westophiles” in the early twentieth century.^51^

Following the fall of the monarchy and the shock of the Treaty of Trianon (1920) when Hungary lost two thirds of its territory, however, there was general distrust of “Western European” politics. The movement known as “Turanism”, which rose to significance between the two world wars, proclaimed an opening to the East and focused on demonstrating the nation’s “Eastern origins” [44]. Hungary’s inglorious role in World War II, caused partly by the desire to recover lost territories, and the Soviet occupation that followed were further major traumata within a span of thirty years.

The Soviet period was perceived as an imposed swing to the East—but not to the “Eastern relatives”. The conscious shaping of a Soviet identity met resistance not only due to its ideology but also to cultural differences. Hungarian intellectuals still defined themselves as “Europeans”, but felt cut off from Western Europe. After the euphoria of the 1989 regime change, hope arose of reintegration into Europe. As a leading Hungarian economist wrote on the prospect of the country’s EU accession, “After several centuries of peripheral existence Hungary would eventually become fully integrated. The significance of this can perhaps only be paralleled with the founding of the state [1000 CE]. We can take roots in Europe, and deep ones now, for a second time”.^52^ The “return to Europe”, however, was also expected to be “painful and slow” [32], and semi-peripheral existence was understood as a frame for the reintegration process [45–50].

As Western European retailers flooded the Hungarian market and Western European industrial giants moved parts of their manufacturing to Hungary (and to other CEE countries) following the 2004 accession, disappointment and euroscepticism followed [51–53]. The perception of the past almost two decades has been that Hungary is only needed as a market and a pool of cheap labour, and the Habsburg image of the country being reduced to a “German colony” has returned. As a result of the above, Hungary’s relations with the EU have necessarily been contradictory: on the one hand there is a deep sense of historical and cultural belonging to (Western) Europe; on the other hand there is an inferiority complex, fear of a loss of identity and a sense of being taken advantage of by core EU countries.

#### Hungary-China Relations

The core-periphery structure seems helpful in explaining the dynamic of Hungarian China-policy. At the same time, the East versus West binary is just as dominant in the background, adding to the instability and constant struggle that characterises the periphery. In 2006 Rumford wrote, “Modernity has seen Eurasia divided according to two principles which have produced a familiar pattern of bordering: an East–West divide, and a core–periphery relation. Neither of these divisions dominates Europe–Asia relations at the present time, although they both still exert an influence over political orientations and continue to inform identity politics” [54]. We argue that these divisions seem to play a more critical role in shaping relations between China and individual EU member states these days than was to be presumed after the optimistic moment of the 2004 EU enlargement.

As recently as 2018 a Hungarian analyst wrote, “It might be expected that China has become an important factor in Hungarian domestic politics, and opposition parties might try to denounce the government as pro-China or pro-communist. In fact, the opposite is true. […] Prominent politicians in the opposition parties barely mention China at all in their public statements, and, unlike in some other countries, its increased political and economic presence has not triggered any alarm in Hungarian political circles or among the wider public. Critical commentary on Hungarian China-policy currently exists only in independent media” [55]. As we will see, the situation has changed, and one of the very first indicators of this change is the case of the Chinese COVID-19 vaccine.

Politicisation of the Chinese vaccine in Hungary can best be understood within the broader context of Hungary-EU and Hungary-China relations. We have already introduced the first, so let us offer a glimpse into the latter as well before moving to the vaccine case study. For the sake of conciseness we summarise the past twenty years only briefly. The first Orbán government (1998–2002) positioned itself strongly against Communist China. Orbán himself received the Dalai Lama in 2000 as Prime Minister, and the official party website of fidesz.hu listed a link to the Free Tibet blog. The next eight years saw a Socialist government (prime ministers: Péter Medgyessy 2002–2004, Ferenc Gyurcsány 2004–2010) which was very China-friendly. As Gyurcsány said in a 2007 interview, “Hungary opposes any form of Taiwanese independence and shares the one China principle.”^53^ Within the first three years of Gyurcsány being PM (2004–2007), sixteen Hungarian ministers visited China, four of them twice, with the PM himself paying two visits.^54^ Throughout the Gyurcsány regime, Orbán-led Fidesz, then in opposition, was fiercely fighting against the government – and against China. “We call on the Hungarian government to use its influence to act in the appropriate fora of the European Union in order to end ethnic violence in China”, said Zoltán Balog, a leading Fidesz politician, in 2009.^55^

However, a mere two months before coming to power in April 2010, Orbán as Fidesz chairman held a state of the nation speech in which he eloquently outlined his future foreign policy. The speech set the tone for the “Opening to the East” policy that was to start the same year, essentially right after Orbán’s coming to power. Here he used the metaphor that became a recurrent image in his rhetoric: “although [Hungary] sails under a Western flag as an EU member state, the wind of the world economy blows from the East.”^56^

One might argue that the severe blow of the global financial crisis to core EU states (with Germany entering a recession and spill-over effects in Hungary) and China’s rapid ascent created a new global setting that Orbán felt the need to accommodate. Under these conditions, China as a rival global centre had (and has) definite leverage for Hungary (and apparently for several CEE states—see the “CEE-China Initiative” launched in 2012—although the domestic political landscape of other CEE states might differ). “Approaching the 2010 elections, it was suggested in professional circles that after a possible change of government, the second Orbán government would pursue a more distant, politically critical, less economically cooperative China-policy. This would have been a sharp reversal compared to the convergence processes of previous years” [56]. In fact, the very opposite happened.

### Framing Hungary-China Relations and Chinese Vaccine-Policy in Hungary

We will briefly introduce three frames that have dominated recent government discourse; then two „counter-frames” employed by the opposition. While our focus is the Chinese vaccine, we will also show that the current frames are deeply rooted in the broader context of how the Fidesz government sees Hungary-EU and Hungary-China relations.

#### Frame 1: Disadvantaged Hungary

The “Disadvantaged Hungary” frame interprets Hungary as a small, subaltern country on the periphery of Europe having to fight for survival. Its enemy is either the “West” (i.e. the core EU states) or, rarely, the “East” (i.e. Russia). If it is the “West”, the fight is emotionally supported by the vision that Hungary might soon outdo all those who have oppressed it this—so it often appears in combination with the second frame (“declining West, rising CEE”). Apart from assuming an economic context, it also often serves to frame texts in which being disadvantaged is personalised, and dramatically so—such as employees of Western companies working for a fraction of the salaries in Western Europe or people having to consume poor-quality food products.

The concept of a “two-speed Europe” is regularly posed as a binary of empowerment (of the “centre”) and disempowerment (of the “periphery”). As Orbán said at the time of the “dual-quality food” scandal, “If we see that there are double standards, and there is a two-speed Europe, we think Central Europeans will certainly be on the receiving end” (Oct. 2017).^57^ He himself employs the “Disadvantaged Hungary” frame regularly to rationalise Hungary’s fight in a malign political environment: “Threats approaching from both the East and the West. […] [A]s we saw that the paths marked out for us by Brussels and Washington were not viable, we were forced to create a new one” (Feb. 2020).^58^ This rejection of a “two-speed Europe” seems to span the entire political spectrum in Hungary, from the far left^59^ to the far right.^60^

The government (Gergely Gulyás, Minister heading the Prime Minister’s Office) first talked publicly about “negotiations” for the Russian and Chinese (Sinopharm) vaccines on 22 October,^61^ which was then reinforced factually by Orbán a week later, “because they’re slightly ahead in vaccine development”.^62^ On 5 November, however, State Secretary for Information and the International Representation of Hungary (Ministry of Foreign Affairs and Trade) Tamás Menczer said in a Facebook video post, “If the antidote is found sooner in the East, then neither the Brussels lobby nor the pharmaceutics lobby can stop us bringing the vaccine to Hungary. The health of the Hungarian people comes first!”^63^ Here he places the Chinese vaccine into the frame of “Disadvantaged Hungary” having to fight for its most basic rights (the health of its own people) against its two enemies: Brussels and the pharmaceutical companies.

At around this time the oppositional media started addressing the issue of vaccine safety and lack of transparency,^64^ providing a platform for the opinions of Hungarian virologists. Orbán, however, continually chose to reframe the discourse as that of “Disadvantaged Hungary”: “[I]t’s best to have access to as many types of vaccine as possible. We mustn’t turn this into a political issue. But there are some who do, who want to wage a new Cold War, playing’East’ against’West’.”^65^ The image of Brussels (“some”) waging an ideological war against pragmatic Hungary (wanting to stop Hungary from purchasing the Chinese vaccine) has remained a central element in vaccine-related discourse by the Hungarian government, as the quotations also illustrate it:

[O]ne cannot allow Hungarians to die, simply because Brussels is too slow in procuring vaccines (22.01.2021).^66^

Here in Hungary we can hardly afford to reject a [Chinese] vaccine which is being used to inoculate Hungarians in Vojvodina. […] One should not use the issue of vaccines in order to prove one’s allegiance to America or to Brussels (15.02.2021).^67^

[T]he lives of the Hungarian people must be saved, and the lives of the Hungarian people are only important for us (19.02.2021).^68^

In the head of a bureaucrat, money is obviously more important than time; but in real life, outside the Brussels bubble, matters are the other way around. […] [W]e have more faith in ourselves than in the Commission (26.02.2021).^69^

#### Frame 2: Declining Western (Core) Europe and Rising Central and Eastern Europe

The perception of a “West” in decline has become a central element in the second and third Orbán government’s discourse on the EU. This is clearly demonstrated by Orbán’s utterances such as “the situation is that the torch of those we could follow has gone out. […] the nucleus of the new European economic era that is just emerging will be not Western but Central Europe” (July 2011)^70^; “the time has almost come for Central Europe to truly be the middle of Europe and evolve into the motor driving the impetus of European economic growth” (May 2014)^71^; “Europe is us, and we do not have to measure up to the tired Brussels elite, who will soon be disillusioned even with themselves. We used to think that Europe was our future; today we know that we are the future of Europe. […] the Carpathian Basin radiates strength” (Feb. 2020)^72^; “[t]he growth centre of the entire European Union is shifting eastwards, to Central Europe” (Oct. 2020).^73^

As the texts reveal, centre and centrality play a very important role in the way Orbán positions his country/region in relation to Western Europe. Instead of postulating commonality, i.e. the sharing of a single European space, he divides the EU into centre and periphery and assumes the role of a peripheral agent trying to instigate a major power shift. This “periphery” versus “centre” (also often referred to simply as “us” and “them”) dichotomy forms the axis of the arguments, and the emotionally loaded images legitimise movement of the centre (domination, power) to Central (Eastern) Europe. The past is allocated to the centre (Western Europe/Brussels), while the future to the periphery (Central and Eastern Europe), making the desired power shift a compelling utopian image.

The oppositional Hungarian Socialist Party declares in its 2014 electoral programme, “Instead of suffering from the renewal of Europe, from the creation of a common new Europe, we want to shape it, so that Hungary should not belong to the periphery, but to the European centre.”^74^ LMP, Hungary’s Green Party, calls for “a Europe of the people, not a Europe of capital”^75^ when speaking against Western European companies’ presence in Hungary. Both the Socialist Party and the Green Party perceive the differences (also the “centre-periphery” dichotomy) in a very similar way as Fidesz (Orbán’s party), but see the solution in moving closer to the centre or levelling things out instead of proclaiming a shift of the centre towards the CEE region.

The vaccine procurement offered ample opportunity for the “Declining Western (Core) Europe and Rising Central and Eastern Europe” frame:

For me the most inspiring example is Serbia, […] those who are outside and have taken care of their own affairs are doing well. […] [L]et’s procure vaccines from elsewhere, knowing that Brussels will either succeed or they won’t. But we can’t afford to stand only on that one Western leg (29.01.2021).^76^

I repeat: for the Government the vaccine is not a political issue. As far as we’re concerned, it doesn’t matter whether the cat is black or white, as long as it catches mice (22.01.2021).^77^

Most interestingly, Orbán is applying Deng Xiaoping’s famous metaphor of “black cat—white cat” to place pragmatism ahead of loyalty.

In mid-January, State Secretary for Information and the International Representation of Hungary (Ministry of Foreign Affairs and Trade) Tamás Menczer was already talking about fierce competition in a victorious tone: “Whether Europe needs the Chinese vaccine or not is well illustrated by the fact that after Thursday’s announcement about advanced negotiations by the Hungarian government, 14 European countries contacted the Chinese manufacturer within a few hours. By now, all of Europe is probably queuing up in China, again.”^78^ Orbán also spoke of the “highest inoculation rate in the entire European Union” (26. 02. 2021).

#### Frame 3: China as Opportunity

Orbán sees a paradigm shift in the world: “Meanwhile China’s gain on global economic weight has been unbroken. […] Consumer welfare societies in the West are over.” (July 2011)^79^; “The engine room of the global economy is no longer in the West, but in the East. […] And it has become increasingly offensive that a few developed countries have been continuously lecturing most of the world on human rights, democracy, development and the market economy. Everyone has had enough of this; and of these the Chinese are the strongest—so they’ve launched another direction of movement, which is called ‘One Belt, One Road’. This is specifically built on mutual acceptance: there is no teacher and no student” (May 2017).^80^ He also declares his complete independence from the EU in China-related matters: “We do not accept restrictions of any kind on cooperation between China and our region” (Oct. 2016).^81^

In a similar vein the Chinese vaccine was positioned as the one coming to rescue Hungary. In mid-January Orbán expressed high hopes for the Chinese vaccine and added a timeline: “Once the health authority completes its job and we’re also able to use the Chinese vaccine, then we’d be able to vaccinate people at a rate that would enable us to get our old lives back before the summer—or perhaps even well before the summer.”^82^ Within two weeks a new Government Decree (No. 19/2021) was published as an amendment to Government Decree No. 488/2020,^83^ allowing for approval of medicines authorised in the European Economic Area or the United Kingdom without testing in Hungary.^84^ The next day the National Institute of Pharmacy and Nutrition approved the application of the Sinopharm vaccine,^85^ circumventing approval by the European Medicines Agency (EMA) and making Hungary the first EU member state to start using the Chinese product. After this move Hungary became a battleground, with Fidesz (Orbán’s party) and the opposition fighting openly over administration of the vaccine.^86^ As the vaccine’s roll-out began and government media were touting the message that the Chinese vaccine is huge help and “saves lives”,^87^ the opposition was busy employing a counter-frame.

#### Frame 4: China Cannot Be Trusted

This frame too has a history, but one that only goes back a few decades and is largely limited to Hungary’s experience with Chinese products. Chinese presence and investment have not been significant in Hungary thus far, but high levels are anticipated in the future [17]. The biggest infrastructure project, the Budapest-Belgrade railway line to be built with Chinese loans, entered the limelight in conjunction with Hungarian government corruption but the novelty soon faded with many similar corruption cases. The Hungarian people are not supportive of China, but they don’t see an enemy or competitor in China either. The frame the opposition could rely on was the one to gain the greatest support: lack of trust.

The Budapest district mayors in the Democratic Coalition led by former PM Ferenc Gyurcsány have sent a letter to Minister of Human Resources Miklós Kásler requesting that only EMA-approved vaccines be administered in their districts because they are the ones to be trusted.^88^ The Democratic Coalition also launched a petition for free vaccine choice following reports of the Sinopharm vaccine as the only option.^89^ Gyurcsány has even ventured to insinuate that Orbán was probably given a different vaccine, not the “worse Chinese one lacking European approval”.^90^ The opposition parties seem to agree that no vaccine should be administered without EMA approval or a Hungarian review process. In late January they issued a joint demand for withdrawal of the new governmental decree that made way for the Chinese vaccine.^91^

The Hungarian population has also shown general distrust in the Chinese vaccine. According to a mid-January survey by Publicus Research, only 27% of those who want a COVID-19 vaccine would accept the Sinopharm product, while 63% would reject it. (The yes–no ratio among the same group for Pfizer is 91%–4%, for Moderna 77–15%, and for Sputnik V 35%–55%.)^92^ This is probably the reason why not just Orbán himself but also Hungarian President János Áder and Zsolt Bayer, a journalist and very influential Orbán supporter, have decided to take the Sinopharm jab—and become live promotion for it.

Both government and opposition found an opportunity to blame the other for generating vaccine hesitancy or refusal among the population. As prominent left-wing political psychologist Péter Krekó wrote on *444.hu*, “In order to counter the […] unfortunately high vaccine refusal rate in Hungary, the government should not prioritize the Russian and Chinese vaccines with less transparent testing and a mass roll-out before the end of testing […].”^93^

By contrast, István Simicskó, an MP for KDNP, the small satellite party of Fidesz, declared at a parliamentary plenary session that “the government cannot count on the Left or unfortunately on Brussels either. […] Left-wing politicians […] constantly suggest that people should not trust the Russian and Chinese vaccines. By doing so, they are trying to make people insecure and thereby endangering and risking lives.” To his question posed to State Secretary Tamás Menczer (Fidesz): “Where would we be with vaccine purchases and vaccinations if we could only rely on Brussels and the Hungarian Left?”,^94^ Menczer’s response was very straightforward: “Nowhere. […] What we are doing is correcting the mistakes of Brussels.”^95^

While opposition politicians and media argue for the safety of EU-approved vaccines—and consequently the superiority of the EU—the government’s discourse creates an adversarial image of the “Left” and “Brussels”, and claims superiority over them.^96^

#### Frame 5: Corrupt Business Between China and the Fidesz Government

This frame has become pervasive in oppositional discourse and managed to unite all non-government parties again Fidesz—notably also the far right which has joined forces with the left-wing parties. Here follow two examples:

Péter Jakab, MP and president of Jobbik, Hungary’s largest far-right party,^97^ addressed Orbán in parliament: “You are saying […] if we are worried about our safety then this year again we will rot in our masks and under the restrictions. Mr. Prime Minister, this is […] blackmail. I know very well that blackmail among your buddies is a cool thing. But this is not Russia. Not China. But Hungary, you know? An EU member state. Here in the Western world the job of the government is not to punish the people but […] to ensure free vaccine choice. […] The European Union is giving us all the help we need. […] Mr. Prime Minister, could you for once in your life not blackmail but treat Europe as a partner? Treat Hungarians as partners? Treat the opposition as partners?”.^98^

Anett Bősz, MP for the left-wing Democratic Coalition, addressed State Secretary Miklós Soltész: “It seems you have a significant interest in vaccinating us with the dubious and much more expensive Chinese vaccine instead of a safe Western one, and you are trying to force it on us with propaganda, threats and discreditation of the Western vaccines. My question, Mr. State Secretary, would be what is the business side of this, and especially for whom?”.^99^

The clear binary created by the opposition consists of the dubious Chinese vaccine versus the safe Western one. In trying to find reasons for what seems a completely illogical choice (the Chinese vaccine over the “safe” one), they conclude that it can only be connected with corruption. Jakab insinuates that China (and Russia) are corrupt anyway, and positions the EU and Western world as the converse. What places Hungary in the “good camp” is its membership in the EU. Europe, Hungarian society and the opposition are all on one side fighting the corrupt Fidesz government and its dirty business with China.

#### Summary of the Hungarian Case Study

By placing the issue of the Chinese vaccine into the first three frames (“Disadvantaged Hungary”, “Declining Western (Core) Europe and Rising Central and Eastern Europe” and “China as Opportunity”) of the five discussed above, the Fidesz-government has been aiming to deliver a message that Hungary (and Hungarian individuals) can only survive with strong national sovereignty safeguarded by Fidesz (Orbán).^100^ In order to keep the “us” intact, or even to increase the sense of collective identity among ethnic Hungarians, Orbán needs constant confrontation with the EU leadership. By placing this confrontation within the centre-periphery relationship can he justify his sabre-rattling against Brussels: the first three frames, based on, the centre-periphery binary, can hold and feed public emotions that are necessary to keep Fidesz in power.

The two frames used by the opposition (“China Cannot Be Trusted” and “Corrupt Business between China and the Fidesz Government”) do not place Hungary in conflict with the EU—on the contrary, they place Hungary under the protective wing of what is perceived as “Europe”. A very eloquent example of this is an interview with Gergely Karácsony, mayor of Budapest and joint PM candidate for three left-wing parties in the 2022 elections.

A: Viktor Orbán managed to thrust Hungary into a cold war between the two greatest powers [USA and China], and our head is now stuck between two cymbal plates. I find this a big mistake, a harmful policy.

Q: Where is our place in this cold war?

A: Preferably outside. It is better for Hungary to stay out of such global conflicts, and it is definitely better not to start serving Chinese interests.

Q: Is it possible to opt out as an EU member state?

A: In this conflict the European Union must take a position of course. But it does matter a lot whether we, as a member state, support this [the EU’s] position or another one. In the case of Hong Kong, for example, Hungary’s veto blocked a unanimous decision. With that we left the security zone that protects an Eastern European small peripheral state from conflict.^101^

While belonging is not a choice for the core, for those on the (semi-)periphery it is a constant struggle. Hungary is moving between the poles of “catching up” with the core EU, trying to become a core itself or “balancing it out” by siding partly with another core. We argue that Hungary’s China-policy is largely defined by the dynamic among these possible choices. A perception of instability and the need for struggle—a major characteristic of the semi—set the background for whichever choice is made.

## Conclusions

Our starting point for this study was our observation that for many years, politicians and policy advisors have been calling for a unified China-policy in the EU, which however has yet to materialise. We are opening a new perspective on this phenomenon: We do not simply join the chorus of those insisting on unity among the EU member states in regard to their China-policy, Instead, we suggest to first take a step back and take a close look at the reasons why EU member countries have different outlooks on China in the first place. By showing how different the “Chinese vaccine” is debated in Germany, a core country in the EU, and Hungary, a country on the semi-periphery of the EU, we have offered a small glimpse into spatial-political divisions within the EU. We argue that a comprehensive re-formulation of an EU China-policy must take these fundamental differences of perspectives and needs of each member country into account. We find that the approach of the “European dependency school”, which maps the position of each EU member state along the division of core, semi-periphery, and periphery, is applicable. We used the case of how the political elites and the media present discourse on COVID-19 vaccinations in Germany and Hungary to illustrate divisions between the outlooks of a typical core and semi-typical periphery country.

Germany’s media reports on China’s COVID-19 vaccine are clearly shaped by scepticism and partly by a lack of proportionality. The generally critical attitude can be explained partially as a reaction to increasing human rights abuses and more assertive foreign policy behaviour by the Chinese leadership over roughly the last decade, which has been hotly debated in German media. However, we suggest that the country’s spatial-political positioning at the core of Europe, which among other things ensures a sufficient supply of vaccines of Western origin and also a stable economic relationship with China that cannot be distorted by short term conflicts, have enabled German media and political discourse to be rather distanced and critical vis-à-vis the Chinese vaccine. These attitudes have allowed frames such as “Vaccination Competition as Competition for World Leadership”, “Ruthless China”, “China’s Vaccine Imperialism”, “China Cannot Be Trusted”, and “China’s Public Relations Failure” to become popular in media and political discourse.

Hungarian vaccine politics demonstrates a dire and inward-looking political landscape. Viktor Orbán is actively framing the Chinese vaccine in a way to prove his superiority over Brussels—and gain further domestic political capital. This dynamic is very different from the framing gaining popularity in Germany about Chinese “vaccine imperialism”. Hungary’s eager endorsement of the Chinese vaccine appears to have roots in Hungary’s perceived disempowerment within the EU. Hungary-China relations seem to be instrumentalised so as to support the “Disadvantaged Hungary”, “Declining Western (Core) Europe and Rising Central and Eastern Europe” and “China as Opportunity” frames, which result from Hungary’s peripheral position in the EU. The two counter-frames employed by the political opposition, “China Cannot Be Trusted” and “Corrupt Business between China and the Fidesz Government”, feed on the understanding that Hungary as a non-core state needs support from the core of the EU against sinking into the periphery and falling prey to other influences.

The perception of Hungary on the periphery (in economic, political or social senses) will most probably also govern the country’s foreign policy from the background, even if its consequences in a non-Orbán government might differ from current ones. Germany’s media commentators, operating from the heart of Europe in political, economic and geographic senses, discuss China from a position of strength, stability and confidence, although ignoring the economic ties that are part of the foundation of that strength and stability.

We agree with Petrova that “[t]he East/West divide […] overemphasises non-compliance by East European EU member states in a way that reinforces the view of a uniform East as a second-class EU citizen” [58]. Our claim with this study is not to demonstrate a uniform East or a uniform West. Instead, we wished to highlight the geopolitical positions (relative “Western” and “Eastern” locations and proximities to perceived centre(s)), as well as the highly complex and strained relationship between core and (semi-)periphery that play a role in shaping the China-policies of EU member states.

The past ten years have been characterised by the assertive global presence of China and the end of two decades of a unipolar, US-led world. With the return of bipolarity on the global level, the old East–West dichotomy and the centre-periphery tensions that have played a role in shaping the foreign policies of Hungary (and presumably of other CEE states although in different ways) and Germany are triggering responses in these states that the EU as an institution might not be prepared for. Unless the EU faces its historic burden, i.e. the core-periphery relations among member states in both economic and in cultural-discursive terrains, chances for a sustainable uniform China-policy within the EU will remain meagre.

## Footnotes

1 Cited in: Poggetti, Lucrezia. “One China—One Europe? German Foreign Minister’s Remarks Irk Beijing.” *The Diplomat*. 09.09.2017. https://thediplomat.com/2017/09/one-china-one-europe-german-foreign-ministers-remarks-irk-beijing/ (accessed 24 April 2020).

2 For a more nuanced analysis of the factors that influence China’s foreign policy, which is not the focus of this article, see for example Cai [2] and Semenov and Tsyk [3].

3 The reaction from China to Gabriel’s words was immediate and harsh. One day later, on 31 August, Chinese Foreign Ministry spokeswoman Hua Chunying said she was “shocked” by Gabriel’s statement. “[She said,] the EU was a regional organization of sovereign states, not a sovereign state itself – relations between [non-EU] countries and EU institutions or EU member states developed parallel, the two were not contradictory.” And: “This is an objective reality.’” She then also referred to the amount of “support” China had offered to the integration of the EU. Cited in: Bai Yunyi. 2020. “German Foreign Minister asks China not to ‘divide Europe’—China shocked by the remark.” (“德国外长要中国别 “分化欧洲” 中方: 对此言论感到震惊”, 环球时报*Huanqiu Shibao* [*Global Times*], 31. 08. 2017. (accessed 24 April 2020).

4 Borrell, Josep. 2020. The Coronavirus pandemic and the new world it is creating. *EEAS*, 23 March. Available at: https://eeas.europa.eu/headquarters/headquarters-homepage/76379/coronavirus-pandemic-and-new-world-it-creating_en (accessed 14 April 2020).

5 Indeed, we are aware of the heterogeneity among the CEE countries as it has been vividly addressed by e,g, Song and Pavlićević [4].

6 For an analysis of how China itself uses the narratives around the pandemic to re-shape world order, see Chang [5].

7 Interestingly, Song’s and Pavlićević’ analysis shows clearly that China is not only well aware of the different needs and interests of the EU member states. China also applies in its EU policy an approach, which they designate as “multilayered multilateralism,” to address this variety [4].

8 The two authors used questionnaires about each member country completed by an expert in each country as the data basis for their overview.

9 Czech Republic, Germany under Chancellor Angela Merkel and Poland.

10 The Netherlands, Sweden, Denmark and the UK.

11 Austria, Belgium, Estonia, Ireland, Latvia, Lithuania and Luxembourg.

12 Fox and Godemont include Bulgaria, Cyprus, Finland, Greece, Hungary, Italy, Malta, Portugal, Romania, Slovakia, Slovenia and Spain in this group. France under Chirac is considered part of this group, while under Sarkozy the country was too critical for this category. And Germany under Schröder, a period of particularly close relations between it and China, is also included in this group.

13 The 16 + 1, later 17 + 1, now presumably 14 + 1 format—which we prefer to call the “China-CEE Initiative” and which is beyond the scope of this paper—has also been discussed in core-periphery terms [12–14].

14 Chen argues that China did more than that in Italy: he found that by delivering aid to Italy, China was able to increase its visibility and positive image in the southern European country [15].

15 Weissenbacher has written extensively about the different networks of researchers on development and dependencies that he ascribes to this very heterogeneous “school” (Weissenbacher 2018, Weissenbacher 2019).

16 We use the term theory here for clarity, leaving aside the larger debate as to whether this strand of thought forms a coherent theory (Cardoso, 1977).

17 I.e. West Germany before unification and unified Germany afterwards.

18 Yet it also has to be noted that “for core countries semi-periphery can also mean periphery (this is why we can call Eastern Europe as semi-periphery also the periphery of the European Union)” [21]. Because of this perspective several authors talk of the “periphery” in the case of Hungary, like Weissenbacher [18, 19], e.g. Bruszt and Karas 2020 [22], Bruszt and Langbein 2015 and 2020 [23, 24]).

19 See Salvati [25] for a more general overview of the different EU members states’ responses to the COVID-19 pandemic.

20 See Wang [28] for a detailed analysis on rising nationalism during the COVID-19 pandemic.

21 https://www.genios.de/presse-archiv/ (accessed 24 July 2021).

22 The corpus excluded German-language news portals from Austria and Switzerland to ensure the analysis would primarily reflect the German discourse.

23 For an excellent overview on framing in European politics see e.g. Semetko and Valkenburg (2000).

24 Such as *Ärtzteblatt*, *Deutsche Apothekerzeitung*, *Pharmazeutische Zeitung*.

25 In order of frequency, with most frequent matches first.

26 Pinghui Zhuang. *South China Morning Post* 25.9.2020. Coronavirus: WHO backed China’s emergency use of experimental vaccines, health official says, SCMP, https://www.scmp.com/news/china/society/article/3103121/coronavirus-who-backed-chinas-emergency-use-experimental (accessed on 24 July 2021).

27 Marc Pitzke. *Spiegel online* 20 April 2020 “Das Impfduell”. All German articles retrieved from the Genios database https://www.genios.de/info/ebib-solution on 27 February 2021. Citations from German media are translations by the authors of this article.

28 Maximilian Kalkhof, Pavel Lokshin, Daniel Friedrich Sturm, Christina Zur Nedden. *Bilanz*, 23 October 2020 “Kampf gegen Corona; Warum China seinen Impfstoff lieber im Ausland testet”.

29 Jan Klauth. *Bilanz*, 10 December 2020 “Chinas COVID-19-Vakzin; Heimlich ködert China mit seinem Impfstoff die Welt”.

30 Speech held in the German Bundestag (protocol no. 19/212, on 25 February 2019 (pp. 26,718 f.).

31 Friederike Böge. *Frankfurter Allgemeine Zeitung*, 25 February 2021 “Pekings Impfkampagne stockt”.

32 Fabian Kretschmer. *Rheinische Post* 20 July 2020, Chinas Impfstoff-Forscher sehen sich auf der Zielgeraden; Die Volksrepublik arbeitet bislang an den meisten potenziellen Corona-Mitteln. Teilweise werden Freiwillige schon außerhalb von klinischen Tests injiziert.

33 Steffen Richter. *Die Zeit*, 29 January 2021 “Besser als gar kein Impfstoff; China betreibt mit Sinovac erfolgreich Gesundheitsdiplomatie. Viele Schwellen- und Entwicklungsländer sind darauf angewiesen dabei gibt es Zweifel an dem Impfstoff.”.

34 Maximilian Kalkhof. *Bilanz*, 8 January 2021 “Corona; Chinas Luxusproblem mit seinem Impfstoff“.

35 Steffen Richter. *Die Zeit* 29 January 2021 “Besser als gar kein Impfstoff; China betreibt mit Sinovac erfolgreich Gesundheitsdiplomatie. Viele Schwellen- und Entwicklungsländer sind darauf angewiesen dabei gibt es Zweifel an dem Impfstoff”; Christian Herrmann. ntv 31 January 2021 Aggressive Vakzin-Diplomatie. China erklärt Biontech-Impfung zur Giftspritze.”.

36 Jan Klauth. *Bilanz*, 10 December 2020 “Chinas COVID-19-Vakzin; Heimlich ködert China mit seinem Impfstoff die Welt”, Jan Klauth. Die Welt, 10 December 2020 “Chinas heimlicher Impfstoff; Europa wartet auf sein Vakzin. Der Pekinger Hersteller Sinovac dagegen liefert bereits Millionen Dosen weltweit aus—das hat Kalkül”.

37 Jan Klauth. *Bilanz*, 10 December 2020 “Chinas COVID-19-Vakzin; Heimlich ködert China mit seinem Impfstoff die Welt”.

38 Dana Heide, Siegfried Hofmann and Martin Kölling. *Handelsblatt* 22 February 2021 “Biontech wird zum Spielball im Konflikt zwischen China und Taiwan”, https://www.handelsblatt.com/politik/international/corona-impfstoff-biontech-wird-zum-spielball-im-konflikt-zwischen-china-und-taiwan/26936182.html?ticket=ST-9909894-BF7zTKbvupsfQy93y9XC-ap2 (accessed 24 July 2021); Friederike Böge. *FAZ* 5 June 2021 “Taiwan braucht Impfstoff. Bloß keine Hilfe aus China”, https://www.faz.net/aktuell/politik/ausland/taiwan-braucht-impfstoff-bloss-keine-hilfe-aus-china-17373720.html (accessed 24 July 2021).

39 Written questions with answers from the Federal Government received during the week of 14 June 2021 (Deutscher Bundestag, document no. 19/30798 (p. 40).

40 Jan Klauth. *Bilanz*, 10 December 2020 “Chinas COVID-19-Vakzin; Heimlich ködert China mit seinem Impfstoff die Welt”.

41 Martin Kessler. *Rheinische Post* 13 February 2021 “Der Impf-Imperialismus; Analyse Während die EU-Länder um genügend Impfstoff ringen, verteilen Russland und China ihre Produkte scheinbar großzügig um die Welt. Damit sichern sie sich Einfluss—oft zum Nachteil der Europäer”.

42 Paraphrase of a parliamentary motion by the Parliamentary Group Alliance 90/The Greens. Deutscher Bundestag, document no. 19/28531, Recommendation for a decision and report by the 19th Committee for Economic Cooperation and Development on the Motion by the Parliamentary Group Alliance 90/The Greens—document no. 19/26887—Organising and supporting the global fight against COVID-19 (15 April 2021). Translation by the authors.

43 Friederike Böge. *Frankfurter Allgemeine Zeitung* 25 February 2021 “Pekings Impfkampagne stockt”; Christiane Kühl. *Merkur* 25 February 2021 “Ganzes Land als ’Labor’? Mit Chinas Hilfe: Serbien und Ungarn düpieren EU beim Corona-Impfen—doch es gibt auch Sorgen”; Anna-Sophie Schneider. *Spiegel online* 6 February 2021“So impft die Welt gegen Corona”.

44 Reply by the Federal Government to the minor interpellation by the parliamentary group The Left—document no. 19/27447—COVID Vaccine Supply as a Global Public Good, document no. 19/27862, 23 March 2021.

45 Klaus Geiger. *Bilanz* 23 February 2021. “Impf-Analyse; Der globale Vergleich zeigt, warum Deutschland versagt”.

46 Data provided by https://kreativ.hu/cikk/juzerszkander_2021_januar.

47 Kossuth Rádió had an average of 1,133,000 listeners (i.e. over one tenth of the population) daily between December 2020 and February 2021. Data provided by https://nmhh.hu/cikk/219031/Budapesti_es_orszagos_napi_radiohallgatottsag_2020_december__2021_februar

48 As of the time of writing, right-wing, national-conservative Fidesz is Hungary’s governing party, chaired by Prime Minister Viktor Orbán. The party’s full name is: “Fidesz—Magyar Polgári Szövetség”: Fidesz—Hungarian Civic Alliance.

49 Urbán, Ágnes (ed.). 2019. “Központosított médiarendszer: Lágy cenzúra.” [“Centralized Media System: Soft Censorship.”] *Mérték Füzetek 17.* [*Mérték Booklets no. 17*] Media Monitor Nonprofit Ltd., Budapest.

50 “kompország”.

51 “nyugatosok”.

52 Inotai, András. 1996. “Integráció: lehetőség és vizsga. Beszélgetés Inotai Andrással.” [“Integration: opportunity and test. Interview with András Inotai.”] In: Európai Utas [European Roader] vol. 6, no. 2, p. 3.

53 “Gyurcsány Ferenc miniszterelnökként még lelkesedett Kínáért.” [“As Prime Minister, Ferenc Gyurcsány was enthusiastic about China”] Délmagyar, 30.05.2021. https://www.delmagyar.hu/orszag-vilag/gyurcsany-ferenc-miniszterelnokkent-meg-lelkesedett-kinaert-7575670/ (accessed 30 June 2021).

54 Data from: https://index.hu/belfold/kina9339/

55 “Fordul a jetibőr köpönyeg.” [“Turning the yeti-skin coat”] Index. 25.06.2011. https://index.hu/belfold/2011/06/25/fideszkina/ (accessed 30 June 2021).

56 Orbán, Viktor. 2010. Újjá kell építeni Magyarországot. [Hungary Needs to Be Rebuilt.] 05.02.2010. *Prime Minister’s website 2010–2015.* http://2010-2015.miniszterelnok.hu/beszed/ujja_kell_epiteni_magyarorszagot (accessed 27 January 2021)—this image of a ship was then broadly cited.in Hungarian news media.

57 MTI. 2017. “Orbán Viktor: A nagy nemzetközi cégek átverik a közép-európai fogyasztókat.” [Viktor Orbán: Large international companies are deceiving Central European consumers.“] *Magyar Hírlap*. 13.10.2017. https://www.magyarhirlap.hu/gazdasag/Orban_Viktor_A_nagy_nemzetkozi_cegek_atverik_a_kozepeuropai_fogyasztokat (accessed 21 June 2021).

58 Fábián, Tamás. 2020. “Orbán: Az elmúlt tíz év volt a legsikeresebb az elmúlt száz évben.” [“Orbán: The past ten years have been the most successful in the last hundred years.”] *Index*. 16.02.2020. https://index.hu/belfold/2020/02/16/orban_viktor_2020_evertekelo/ (accessed 21 June 2021).

59 “Orbán Viktor a kétsebességes Európa legnagyobb élharcosa, leszakítja Magyarországot.” [“Viktor Orbán is the biggest supporter of a two-speed Europe, he tears Hungary off.”] 2019. *Lehet más a politika.* [*Website of Hungary’s Green Party.*] 30.01.2019. https://lehetmas.hu/europai-unio/orban-viktor-ketsebesseges-europa-legnagyobb-elharcosa-leszakitja-magyarorszagot/ (accessed 24 February 2021).

60 Nagy, József. 2018. „Vona Gábor: Nem lesz torkosborz.” [“Gábor Vona will not give in.”] 06.04.2018. *24.hu.* https://24.hu/belfold/2018/04/06/vona-gabor-nem-lesz-torkosborz/ (accessed 04 March 2021).

61 Presinszky, Judit. 2020. “Gulyás: Orosz vagy kínai vakcinát is vehet a kormány.” [“Gulyás: The government may buy Russian or Chinese vaccine as well”] *Telex.* 22.10.2020. https://telex.hu/belfold/2020/10/22/kormanyinfo-gulyas-gergely-oktober-22 (accessed 04 March 2021).

62 Orbán, Viktor. 2020. “Prime Minister Viktor Orbán on the Kossuth Radio Programme’Good Morning Hungary’.” 30.10.2020. *Prime Minister’s Website.* http://www.miniszterelnok.hu/prime-minister-viktor-orban-on-the-kossuth-radio-programme-good-morning-hungary-30/ (accessed 18 February 2021).

63 Menczer, Tamás. 2020. “Koronavírus elleni vakcinamintát kaphat a kormány bevizsgálásra Kínából.” [“The government may receive vaccine sample from China for review”] *Facebook.* 05.11.2020. https://www.facebook.com/menczer.tamas/videos/966248520533898/ (accessed 04 March 2021).

64 Bolcsó, Dániel. 2020. “Orosz és kínai vakcinát venne a kormány, de a gyorsaság nagy kockázatot is jelenthet.” [“The government would purchase Russian and Chinese vaccines, but the haste might carry big risks.”] *Telex.* 11.11.2020. https://telex.hu/koronavirus/2020/11/11/orosz-kinai-vakcina-oltas-koronavirus-jarvany-kockazatos-biztonsagos-szijjarto-peter-pfizer-biontech-astrazeneca (accessed 04 March 2021).

65 Orbán, Viktor. 2020. “Prime Minister Viktor Orbán on the Kossuth Radio Programme’Good Morning Hungary’.” 13.11.2020. *Prime Minister’s Website.* http://www.miniszterelnok.hu/prime-minister-viktor-orban-on-the-kossuth-radio-programme-good-morning-hungary-32/ (accessed 18 February 2021).

66 Orbán, Viktor. 2021. “Prime Minister Viktor Orbán on the Kossuth Radio Programme’Good Morning Hungary’.” 22.01.2021. *Prime Minister’s Website.* http://www.miniszterelnok.hu/prime-minister-viktor-orban-on-the-kossuth-radio-programme-good-morning-hungary-38/ (accessed 18 February 2021)—the topic then saw a media debate arguing for or against “the slowness of Brussels”.

67 HÍRTV. 2021. “Orbán Viktor: Magyarország erősebb lesz a járvány után, mint előtte volt.” [“Orbán: Hungary will be stronger after the epidemic than it was before.”] 15.02.2021. https://hirtv.hu/ahirtvhirei/kezdodik-az-orszaggyules-tavaszi-ulesszaka-eloben-a-hirtv-musoran-2516883 (accessed 21 June 2021).

68 Orbán, Viktor. 2021. “Prime Minister Viktor Orbán on the Kossuth Radio Programme’Good Morning Hungary’.” 19.02.2021. *Prime Minister’s Website.* http://www.miniszterelnok.hu/orban-viktor-a-kossuth-radio-jo-reggelt-magyarorszag-cimu-musoraban-36/ (accessed 25 February 2021). Translation by the authors.

69 Haszán, Zoltán. 2021. “A kormány azért nem rendelt többet a Moderna vakcinájából, mert sokallta az árát.” [“The government did not order more of the Moderna vaccine, because they found it too expensive.”] *444*, 28.02.2021. https://444.hu/2021/02/28/a-kormany-azert-nem-rendelt-tobbet-a-moderna-vakcinajabol-mert-sokallta-az-arat (accessed 21 June 2021).

70 hvg.hu. 2011. “Orbán a Nyugat alkonyát, fideszes EP-képviselők legfeljebb szürkületet látnak” [“Orbán sees the twilight of the West, EP members of Fidesz only see late afternoon”] *HVG*, 01.08.2011. https://hvg.hu/itthon/20110801_Nyugat_borton_kenyszerlakhely (accessed 21 June 2021)—the oppositional website mocks Orbán’s idea of the West being a “prison” or “compulsory residence” for Hungarians.

71 WA. 2014. “Európában nem élhetünk más pénzén” [“We cannot live on someone else’s money in Europe”] *Magyar Nemzet*, 08.05.2014. https://magyarnemzet.hu/archivum/kulfold-archivum/orban-europaban-nem-elhetunk-mas-penzen-4061110/ (accessed 21 June 2021).

72 Orbán, Viktor. 2020. “Viktor Orbán’s’State of the Nation’ Address.” https://magyarnemzet.hu/belfold/orban-viktor-evertekelo-beszede-nezze-meg-7785506/*Magyar Nemzet.* 16.02.2020 (accessed 21 June 2021).

73 MTI. 2020. “Orbán Viktor: Felértékelődik Közép-Európa.” [“Viktor Orbán: Central Europe gains in value.”] Világgazdaság. 14.10.2020. https://www.vg.hu/vilaggazdasag-magyar-gazdasag/2020/10/orban-viktor-felertekelodik-kozep-europa (accessed 21 June 2021).

74 Igazság, biztonság, szabadság, jólét. Az MSZP ajánlata Magyarországnak. [Justice, security, freedom, prosperity. The offer of the Hungarian Socialist Party to Hungary] official party publication, October 2013.

75 “Az LMP nyilatkozata Magyarország EU-csatlakozásának 15. évfordulójára.” [“LMP Statement on the 15th Anniversary of Hungary’s Accession to the EU.”] 2019. *Lehet más a politika.* [*Website of Hungary’s Green Party.*] 01.05.2019. https://lehetmas.hu/europai-unio/az-lmp-nyilatkozata-magyarorszag-eu-csatlakozasanak-15-evfordulojara/ (accessed 24 February 2021).

76 Orbán, Viktor. 2021. “Prime Minister Viktor Orbán on the Kossuth Radio Programme’Good Morning Hungary’.” 29.01.2021. *Prime Minister’s Website.* http://www.miniszterelnok.hu/prime-minister-viktor-orban-on-the-kossuth-radio-programme-good-morning-hungary-39/ (accessed 18 February 2021).

77 Czinkóczi, Sándor. 2021. “Orbán: Mindegy, hogy a macska fekete vagy fehér, csak fogja meg az egeret.” [“Orbán: It doesn’t matter whether the cat is black or white, as long as it catches mice”] *444* 22.01.2021. https://444.hu/2021/01/22/orban-mindegy-hogy-a-macska-fekete-vagy-feher-csak-fogja-meg-az-egeret (accessed 21 June 2021).

78 Menczer, Tamás. 2021. “Európa Kínában áll sorban a vakcináért.” [“All of Europe is queuing up in China for the vaccine.”] *Facebook.* 17.01.2021. https://www.facebook.com/menczer.tamas/videos/127598505848377 https://24.hu/belfold/2021/01/17/menczer-tamas-oltas-kinai-vakcina-magyar-tudosok/ No official English translation is available, the translation provided here is that of Á. Révész.

79 Orbán, Viktor. 2011. “Sikeres államot csak akkor lehet felépíteni, ha erős nemzet áll mögötte.” [“A successful state can be built only with a strong nation behind it.”] *Kormányportál 2010–2014.* [*Website of the Hungarian Government 2010–2014*.] 23.07.2011. https://2010-2014.kormany.hu/hu/miniszterelnokseg/miniszterelnok/beszedek-publikaciok-interjuk/fsikeres-allamot-csak-akkor-lehet-felepiteni-ha-eros-nemzet-all-mogotte (accessed 29 January 2021)—a long opinion piece on this was published by Antal. Attila. 2011. „Orbán tusnádi diagnózisa és helytelen következtetései.” [“Orbán’s Tusnád diagnosis and incorrect conclusions.”] HVG. 28.07.2011. https://hvg.hu/velemeny/20110728_antal_attila_orban (accessed 22 June 2021).

80 Orbán, Viktor. 2017**.** “Interview with Prime Minister Viktor Orbán on Magyar Televizió (Hungarian public television) summarising his recent visit to Beijing.” 16.05.2017. *About Hungary.* http://abouthungary.hu/speeches-and-remarks/interview-with-prime-minister-viktor-orban-on-magyar-televizio-hungarian-public-television-summarising-his-recent-visit-to-beijing/ (accessed 24 February 2021).

81 Tóth, Loretta. 2016. “Közép-Európa a jövő reménysége.” [“Central Europe is the hope for the future.”] Magyar Hírlap. 07.10.2016. https://www.magyarhirlap.hu/ajanlo/KozepEuropa_a_jovo_remenysege (accessed 21 June 2021).

82 Orbán, Viktor. 2021. “Prime Minister Viktor Orbán on the Kossuth Radio Programme’Good Morning Hungary’.” 15.01.2021. *Prime Minister’s Website.* http://www.miniszterelnok.hu/prime-minister-viktor-orban-on-the-kossuth-radio-programme-good-morning-hungary-37/ (accessed 18 February 2021)—the phrase “to get our old lives back before the summer” was then widely cited across the Hungarian media landscape.

83 “488/2020. (XI.11.) Kormányrendelet a biztonságos vészhelyzeti gyógyszerellátáshoz szükséges egyes intézkedésekről.” [“488/2020. (XI.11.) Government decree on certain regulations concerning the safety of emergency medication supplies”] *Jogtár.* [*Legal Database.*] https://net.jogtar.hu/jogszabaly?docid=A2000488.KOR&dbnum=1 (accessed 02 March 2021).

84 Two further criteria require them to already be in use in at least three states (at least one of which is an EU member state or candidate) and at least one million people to have already received them.

85 “Országos tisztifőorvos: bővül az oltási paletta” [Chief Medical Officer: Our selection of vaccines has broadened”] 2021. *Central Coronavirus Website.* 29.01.2021. https://koronavirus.gov.hu/cikkek/orszagos-tisztifoorvos-bovul-az-oltasi-paletta (accessed 02 March 2021).

86 Also: “Hungary became the first European country to administer Sputnik V in February 2021” (Leigh 2021).

87 “Kezdődik az oltás a hatásos és biztonságos kínai vakcinával.” [“Roll-out of the effective and safe Chinese vaccine begins.”] *Origo* 24.02.2021 https://www.origo.hu/itthon/20210223-kinai-vakcina-nagy-eredmenyek-segitseg.html (accessed 17 July 2021).

88 “Káslerhez fordultak a DK-s polgármesterek, hogy kerületeikben csak az unió által jóváhagyott vakcinával oltsanak.” [“District mayors of DC request Kásler to exclusively use EU-approved vaccines in their districts”] 2021. HVG. 14.02.2021. https://hvg.hu/tudomany/20210214_DK_polgarmester_vakcina_oltas (accessed 04 March 2021).

89 24.hu. 2021. “DK: Legyen szabad vakcinaválasztás! Fidesz: A Gyurcsány-lista pártjai oltásellenesek.” [“DC: Let’s have free vaccine choice! Fidesz: The parties with Gyurcsány are against vaccination”] *24.hu*. 21.02.2021. https://24.hu/kozelet/2021/02/21/dk-fidesz-oltas/ (accessed 21 June 2021).

90 Német, Tamás. 2021. “Gyurcsány: Miért hinnénk el Orbánéknak, hogy az európai engedéllyel nem rendelkező, kínai vakcinával oltatják magukat?” [“Gyurcsány: Why should we believe Orbán and his people that they get inoculated with the Chinese vaccine that lacks EU approval?”] *Telex*. 01.03.2021. https://telex.hu/belfold/2021/03/01/gyurcsany-ferenc-orban-viktor-kinai-vakcina-csirkepaprikas (accessed 04 March 2021).

91 Csatári, Flóra Dóra. 2021. „Az ellenzék a kínai vakcinának vizsgálat nélkül utat nyitó rendelet visszavonását követeli.” [“The opposition demands to revoke the decree that gave way to the Chinese vaccine without review.”] *Telex*. 29.01.2021. https://telex.hu/belfold/2021/01/29/kinai-vakcina-ellenzek-rendelet-visszavonasat-kovetelik-a-kormanytol (accessed 03 March 2021).

92 “Növekszik az oltási hajlandóság, a keleti vakcinák továbbra is elutasítottak” [“Vaccination acceptance grows, Eastern vaccines remain rejected”] 2021. Publicus Research. 29.01.2021 https://publicus.hu/blog/novekszik-az-oltasi-hajlandosag-keleti-vakcinak-tovabbra-is-elutasitottak/ (accessed 04 March 2021).

93 Krekó, Péter. 2021. “Vakcinapolitika a hazai post-truth laboratóriumban.” “Vaccine politics in the domestic post-truth laboratory.” *444.hu* 04.01.2021 https://444.hu/2021/01/04/vakcinapolitika-a-hazai-post-truth-laboratoriumban (accessed 15 July 2021).

94 Simicskó, István. 2021. “Where would we be with vaccine purchases, if we waited for Brussels and the Hungarian Left?” *Hungarian National Assembly* 16.03.2021, A/15438 question. https://www.parlament.hu/web/guest/felszolalasok-keresese (accessed 15 July 2021).

95 Menczer, Tamás. 2021. “Where would we be with vaccine purchases, if we waited for Brussels and the Hungarian Left?” *Hungarian National Assembly* 16.03.2021, A/15438 answer to the question. https://www.parlament.hu/web/guest/felszolalasok-keresese (accessed 15 July 2021).

96 Due to the Fidesz government’s regular denunciation of Brussels a new verb has seen widespread use in oppositional political discourse over the past few years: “to brussels”—meaning to condemn EU institutions.

97 Jobbik has shifted towards the centre over recent years, losing much of its radicalism but gaining a moderate electorate. It has 13.1% of the current parliament.

98 Jakab, Péter. 2021. *Hungarian National Assembly* 15.02.2021, remark on the pre-agenda address. https://www.parlament.hu/web/guest/felszolalasok-keresese (accessed 15 July 2021).

99 Bősz, Anett. 2021. “The government’s lie has no legs.” *Hungarian National Assembly* 01.03.2021, A/15239 question. https://www.parlament.hu/web/guest/felszolalasok-keresese (accessed 15 July 2021).

100 For a thoughtful discussion about the meaning of sovereignty in and after the pandemic, see Makarychev and Romashko [57].

101 Tóth, Csaba Tibor and Petróczi, Rafael. 2021. “Karácsony Gergely: Ma valószínűleg az EU nehezebben venné fel Magyarországot, mint Szerbiát.” [“Gergely Karácsony: Today the EU would be more reluctant to admit Hungary than Serbia”] https://azonnali.hu/cikk/20210624_karacsony-gergely-ma-valoszinuleg-az-eu-nehezebben-venne-fel-magyarorszagot-mint-szerbiat (accessed 17 July 2021).
